# New Insights into the Use of Liraglutide—Impact on Cardiovascular Risk and Microvascular Outcomes

**DOI:** 10.3390/biomedicines11041159

**Published:** 2023-04-12

**Authors:** Magdalena Wronka, Julia Krzemińska, Ewelina Młynarska, Jacek Rysz, Beata Franczyk

**Affiliations:** 1 Department of Nephrocardiology, Medical University of Lodz, ul. Zeromskiego 113, 90-549 Lodz, Poland; wronkam96@gmail.com (M.W.); bfranczyk-skora@wp.pl (B.F.); 2 Department of Nephrology, Hypertension and Family Medicine, Medical University of Lodz, ul. Zeromskiego 113, 90-549 Lodz, Poland; jacek.rysz@umed.lodz.pl

**Keywords:** liraglutide, glucagon-like peptides-1, microvascular, cardiovascular risk, type 2 diabetes mellitus

## Abstract

Despite the availability of many glucose-lowering drugs, patients with type 2 diabetes mellitus (T2DM) often do not achieve the desired effect, and cardiovascular complications remain the leading cause of death in this group of patients. Recently, more and more attention has been paid to the properties of drugs, with particular emphasis on the possibility of reducing cardiovascular risk. One of them is liraglutide, which belongs to long-acting analogs of glucagon-like peptides-1 (GLP-1); it imitates incretins and causes an increase in insulin secretion. The current study focused on analyzing the efficacy and safety of liraglutide, as well as its impact on microvascular and cardiovascular outcomes in the treatment of patients with T2DM. Hyperglycemia-induced endothelial dysfunction, which is known to play a key role in maintaining cardiovascular homeostasis, is common in diabetes. Liraglutide reduces endothelial dysfunction by reversing damage to endothelial cells. By reducing the generation of reactive oxygen species (ROS), thereby affecting Bax, Bcl-2 protein levels, and restoring signaling pathways, Liraglutide reduces oxidative stress, inflammation, and prevents endothelial cell apoptosis. Liraglutide has beneficial effects on the cardiovascular system; patients with high cardiovascular risk particularly benefit from treatment, as it reduces their major adverse cardiovascular event (MACE) rate, which takes into account cardiovascular death, stroke, and non-fatal myocardial infarction. Liraglutide reduces the occurrence and progression of nephropathy, which is one of the most common microvascular complications of diabetes.

## 1. Introduction

**Figure 1:**
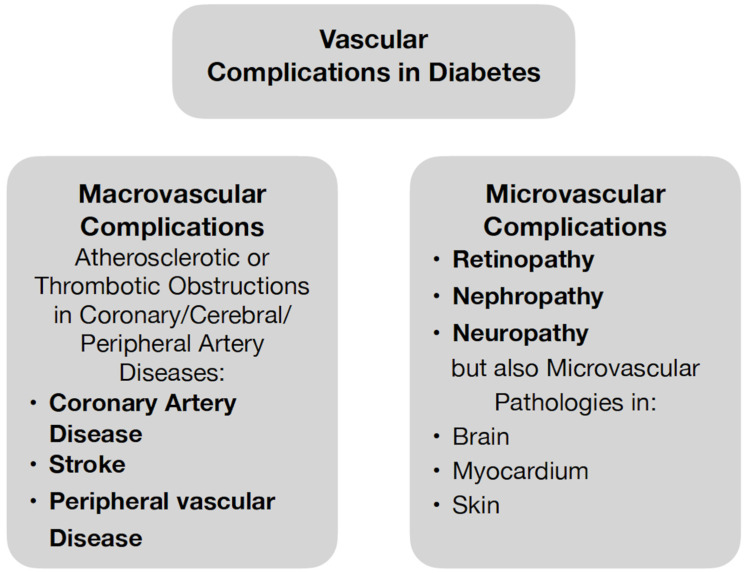
Vascular complications in diabetic patients [4,5].

Diabetes is one of the most common disease entities today [1]. It represents a major burden on the health care system [2]. Diabetics have a higher cardiovascular risk [3]; macrovascular complications are considered the most important cause of death in this group of patients [4]. However, all vascular complications are essential (Figure 1) [4,5].

Glucagon-like peptide 1 (GLP-1) is an incretin, a polypeptide hormone that is secreted from the upper gastrointestinal tract as a result of food consumption [6,7]. It causes an increase in insulin secretion by binding to pancreatic islet B receptors. Its elimination involves the cleavage of peptides by a specific enzyme, dipeptidylpeptidase-4 (DPP-4). Due to its properties, GLP-1 has become an important mediator in the treatment of type 2 diabetes mellitus (T2DM) in the form of drugs (analogs of this incretin) [6]. There are six drugs included in this group; they can be divided into short-acting and long-acting [8,9]. They are presented in Table 1 [8,9].

Renal microvascular dysfunction increases the risk of death in diabetics from cardiovascular causes [10,11]. Moreover, diabetes is the most common cause of chronic kidney disease [10]. Likewise, diabetic neuropathy increases patient mortality. However, it is worth noting that microvascular complications also affect pathologies other than nephropathy, retinopathy, and neuropathy. It has been observed that microvascular dysfunction also affects the brain, causing changes comparable to dementia [5] or leading to diabetic cardiomyopathy [4].

Microvascular complications increase the risk of myocardial infarction (MI), stroke, and cardiovascular death [11,12]; a 1% increase in glycated hemoglobin (HbA1c) results in a 7.5% increase in cardiovascular mortality in patients with T2DM [4]. Thus, it is essential to prevent or delay the onset of diabetes complications. For this purpose, a cardiovascular risk-reducing agent such as liraglutide [11] is worth adding to treatment based on control and maintenance of normal glycemia and reduction of oxidative stress [5].

Cardiovascular and all-cause mortality is higher in patients with T2DM than in non-diabetic patients. As such, a focus on preventing cardiovascular complications in this group of patients is essential [13,14]. The basic premise of the treatment of T2DM is the use of glucose-lowering drugs; however, due to the very high risk of adverse cardiovascular events, attention has recently focused on the prevention of cardiovascular events and diseases [15]. Despite the broad spectrum of hypoglycemic drugs, maintaining desired glycemic levels while protecting against hypoglycemic episodes and maintaining body weight can be problematic. GLP-1 receptor agonists address these needs not only by lowering HbA1c levels, thereby reducing cardiovascular risk, but also by reducing body weight and preventing hypoglycemia [7].

## 2. General Information about the Group of GLP-1 Receptor Agonists with Special Emphasis on Liraglutide

Liraglutide is one of the long-acting GLP-1 receptor agonists. It is produced by recombinant DNA and is a drug administered by daily subcutaneous injections, as it has poor bioavailability when administered orally [6,8,16]. To prevent its degradation by DPP-4, it is combined with a 16-carbon fatty acid during formulation to protect the cutting site [8]. The indication for its use is glycemic control in patients with T2DM in combination with a healthy lifestyle, with or without other glycemic-lowering drugs [6,8]. Since metformin in T2DM is the first-line treatment, GLP-1 analogues, and therefore liraglutide, are used when metformin is intolerant or contraindicated and when the target HbA1c level has not been achieved within 3 months or HbA1c is more than 1.5% above the target threshold. Large doses of liraglutide have also been approved for the treatment of obesity or overweight with comorbidities, and weight loss can reach 2.9 kg [16]. The advantage is that it can be used at any time of day, injected subcutaneously once a day, and is unrelated to food intake [8]. Therapy is started with a dose of 0.6 mg for a week then increased to a maximum dose of 1.8 mg [6,8].

The mechanism of action of GLP-1 receptor agonists is glucose dependent and involves an increase in insulin secretion, inhibiting glucagon secretion, and thereby lowering glucose levels. Additional effects of short-acting GLP-1 receptor agonists include inducing satiety, suppressing appetite, and decreasing post-meal glucose values due to delayed gastric emptying [8,9]. In addition, these drugs increase the proliferation of pancreatic B cells. The mechanism of action of these drugs makes them not prone to cause hypoglycemia unless they are used together with insulin-enhancing drugs or insulin, in which case the risk increases [8].

HbA1c levels are lowered by these drugs in the 1–2% range, with long-acting preparations showing better results, just as they have a better effect at lowering fasting glucose. However, post-meal glucose spikes are better controlled with short-acting drugs [8,9]. GLP-1 receptor agonists reduce cardiovascular risk by reducing atherosclerotic complications. Another beneficial effect is their ability to reduce body weight; high dose liraglutide and semaglutide are approved drugs for the treatment of obesity. In addition, this group of drugs lowers systolic blood pressure and has a beneficial effect on the lipid profile, reducing total cholesterol (TC), triglyceride (TG), low-density lipoprotein cholesterol, and increasing high-density lipoprotein cholesterol [8].

**Figure 2:**
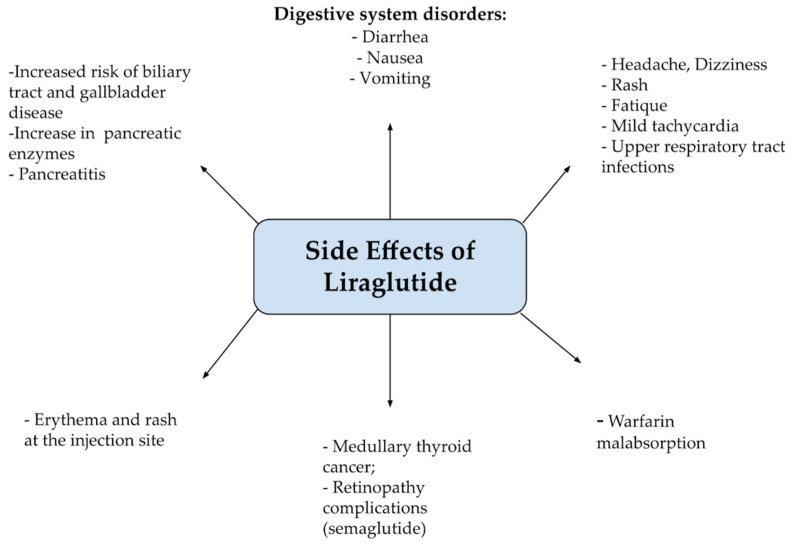
Most common adverse events of liraglutide [6,8,9,16].

The most common side effects of treatment are gastrointestinal symptoms, including diarrhea, nausea, and vomiting, as well as other side effects such as headache and dizziness, rash, fatigue, mild tachycardia, and upper respiratory tract infections. However, these subside with time and can be prevented by gradual dose intensification [6,8,9]. Particular attention should be paid to gastrointestinal symptoms, as significantly increased symptoms can lead to dehydration and acute kidney damage [8,16]. GLP-1 receptor agonists have been shown to increase the risk of biliary tract and gallbladder disease; they also cause an increase in pancreatic enzymes. Because of the route of administration, erythema and rash may appear at the injection site; in the case of exenatide, nodules may also appear. There have been suspicions that the treatment in question may contribute to the development of medullary thyroid cancer; however, these concerns have not been confirmed. There are also concerns that GLP-1 agonists may increase the risk of retinopathy complications, especially semaglutide, as shown in the SUSTAIN 6 study; however, confirmation of these speculations requires further studies [8]. It is worth noting that patients should be monitored for international normalized ratio values when using warfarin, as GLP-1 analogs may alter its absorption [16]. Figure 2 presents the most common adverse events during therapy with GLP-1 analogs [6,8,9,16].

Pregnant patients and those with hypersensitivity to GLP-1 receptor agonists should not use drugs from this group [16]. GLP-1 analogs should be used with caution in patients with severe kidney disease. Patients with an estimated glomerular filtration rate (eGFR) less than 45 mL/min/1.73 m^2^ or creatinine clearance <30 mL/min should not use exenatide; those with end-stage renal disease should not use exenatide and lixisenatide. Severe gastrointestinal disease and gastroparesis are also among the contraindications of this drug. Patients with a high risk of or history of hemorrhagic or necrotizing pancreatitis should avoid GLP-1 receptor agonists; a history of medullary thyroid cancer or multiple endocrine neoplasia type 2 syndrome is also a contraindication to therapy [8,9]. Due to cardiovascular conduction abnormalities, including sinus tachycardia, dulaglutide should be administered with extreme caution to patients with a history of arrhythmias. Exenatide can cause thrombocytopenia, so once confirmed, the drug should be discontinued. Semaglutide can cause retinopathy [9].

## 3. Safety and Efficacy of Liraglutide in the Treatment of Type 2 Diabetes

The purpose of the study by Htike et al. [7] was to evaluate the safety and efficacy of GLP-1 analogs in patients with T2DM. It was observed that all GLP-1 analogs resulted in lowering both HbA1c and fasting blood glucose with the best results in patients taking dulaglutide (1.21% reduction in HbA1c) and liraglutide (1.15% reduction in HbA1c). The long-acting formulations of liraglutide, dulaglutide, and exenatide, given once a week, were shown to have better results in lowering HbA1c when compared to the short-acting drugs. However, aside from the greater effectiveness of glycemic control on other factors, such as body weight, blood pressure, and lipidogram values, no significant differences were noted. An increased risk of hypoglycemic episodes was observed during therapy, excluding abiglutide. An increased incidence of diarrhea, nausea, and vomiting among all drugs was also reported; however, once administered, exenatide has a lower incidence of nausea and vomiting [7]. A meta-analysis by Taheri et al. [17] compared liraglutide administered once daily with dulaglutide administered once weekly in terms of safety and efficacy in the treatment of T2DM. During the study, a greater reduction in HbA1c (10%) was observed in patients taking dulaglutide when compared to liraglutide. However, the risk of gastrointestinal side effects was higher in the dulaglutide group. It is worth noting that there were no statistically significant differences in fasting glucose levels, hypoglycemic episodes, or serious adverse events. Taheri et al. [17] emphasize that a limitation of their meta-analysis is the small number of included studies, so further research on this topic is needed [17]. A randomized controlled trial (RCT) by Seino et al. [18] evaluated the safety and efficacy of liraglutide administered at a dose of 1.8 mg in Japanese patients with T2DM who were already being unsuccessfully treated with a dose of 0.6 mg. Over the course of the 26 week study, better glycemic control was noted with the higher dose of liraglutide, causing a further decrease in HbA1c in these patients when compared to lower doses. Use of liraglutide in general was associated with weight reduction, but increasing the dose to 1.8 mg provided little benefit. Similarly, despite the dose increase, no increased risk of hypoglycemia or side effects was observed. Both doses were well tolerated by patients [18]. Wang et al. [19] focused on a short-term evaluation of the safety and efficacy of liraglutide in patients with T2DM and coronary artery disease (CAD). Compared to metformin monotherapy or treatment with other hypoglycemic drugs, liraglutide monotherapy or its combination with other glucose-lowering drugs significantly improved indices of glycemic control, indices of cardiac function, and resulted in improvements in lipidogram. Moreover, liraglutide therapy showed a positive safety profile, as it did not increase the risk of hypoglycemia and gastrointestinal side effects. Thus, it appears that liraglutide may be a beneficial cardiovascular protective drug in patients with T2DM and established CAD; however, the results of this study were based on a small number of studies and had several limitations, so conclusions regarding safety should be treated with caution [19]. The SUSTAIN 10 trial was conducted to compare the efficacy and safety of a once-weekly dose of 1.0 mg of semaglutide with a once-daily dose of 1.2 mg of liraglutide in patients already taking one to three oral antidiabetic drugs. Over 30 weeks, semaglutide showed a greater reduction in HbA1c and FPG levels when compared to liraglutide. The percentage of patients achieving target HbA1c <7% or <6.5% was also higher in the semaglutide group. The semaglutide group showed a mean weight loss of −5.8 kg when compared to −1.9 kg in the liraglutide group; the percentage of patients achieving the weight loss target of 5% or 10% was also higher in the semaglutide group. The semaglutide group achieved better results in lowering TC and TG. Regarding adverse events of treatment, a higher number of gastrointestinal disorders (most commonly nausea) were recorded in the semaglutide group (70.6%) when compared to the liraglutide group (66.2%). However, more serious adverse events were recorded in the liraglutide group. There were isolated cases of patients with acute pancreatitis in the liraglutide group and retinopathy in both groups. Overall, it was shown that both therapies were fairly well tolerated by patients [20]. A comparison of the studies described above is shown in Table 2 [7,17,18,19].

## 4. Molecular Effects of Liraglutide Treatment—Effects on Oxidative Stress and Endothelial Dysfunction

The endothelium is an important regulatory and endocrine organ; endothelial dysfunction underlies many cardiovascular diseases (CVD) [21]. Studies have shown that hyperglycemia is one factor that damage the endothelium [22,23]. Elevated glucose concentration causes, among other things, generation of reactive nitrogen species, increase in the concentration of Bax protein, leading to apoptosis of endothelial cells of pancreatic islets through activation of Jun N-terminal kinase [22].

A study by Le et al. [24] was conducted to examine the mechanism of the protective properties of liraglutide against endothelial cells. For this purpose, a cell line derived from mouse islet microvascular endothelial cells was used. Since islet microvascular endothelial cells are injured in T2DM, the study used palmitic acid as a model of injury; this caused an increase in reactive oxygen species (ROS) and promoted cell apoptosis. It has been proven that liraglutide therapy reduces microvascular endothelial cell damage by reversing the induced damage. This occurs as a result of the mechanism of reduction of oxidative stress and apoptosis due to an increase in the concentration of anti-apoptotic protein Bcl-2 and a decrease in the level of pro-apoptotic protein Bax. There is an increase in the expression and secretion of endothelin-1, which is a stimulant of insulin secretion in β-cells. The beneficial effects of liraglutide also include restoration of glucagon-like peptide-1 receptor/phosphorylated protein kinase A (GLP-1R/PKA) and guanosine 5′-triphosphate cyclohydrolase 1/endothelial nitric oxide synthase (GTPCH1/eNOS) pathway signaling responsible for the drug’s protective role. 

**Figure 3:**
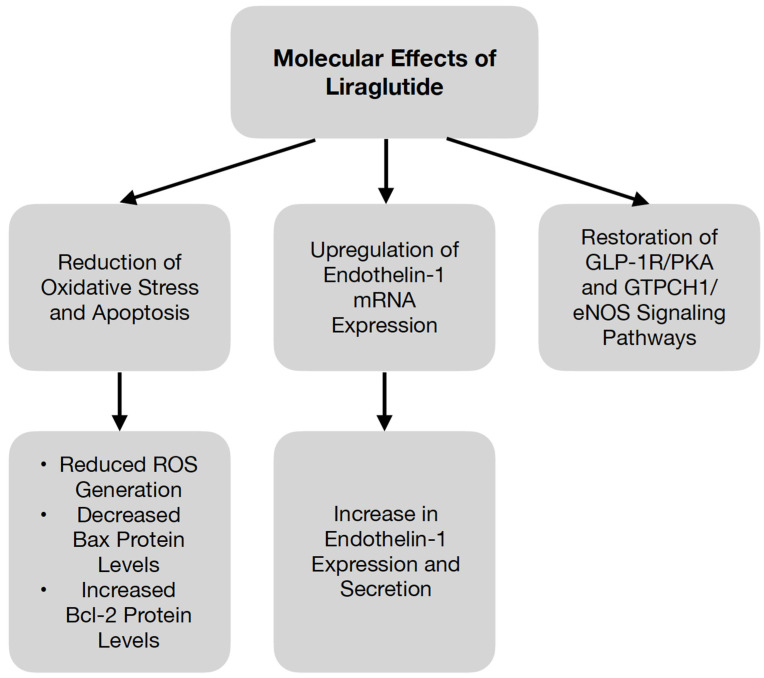
Molecular effects of liraglutide [24].

Molecular effects of liraglutide are shown in Figure 3 [24].

A study by Zhang et al. [25], which investigated the protective role of liraglutide against cardiac microvascular endothelial cells, provides a consistent finding. Liraglutide via the PI3K/Akt/survivin pathways appeared to restore homeostasis after hypoxia/reoxygenation-induced oxidative damage, which was the cause of cell apoptosis due to Ca^2+^ overload. It was observed that liraglutide influenced calcium metabolism by decreasing Ca^2+^ concentration through reduced expression of inositol trisphosphate receptor, which is responsible for its release from sarcoplasmic reticulum (SR), and increasing Ca^2+^ uptake into the SR via effects on SERCA2a.

Studies on patients have also confirmed that liraglutide has a beneficial effect on oxidative stress, lowers inflammatory markers, and counteracts endothelial dysfunction [26,27,28]. In a study by Rizzo et al. [26], liraglutide was added to metformin therapy. They observed, among other things, a decrease in markers such as lipid hydroperoxides and heme oxygenase-1, indicating a reduction in oxidative stress. On the other hand, the increase in glutathione concentration is the result of a decrease in the ratio of oxidized glutathione to total glutathione and demonstrates the antioxidant capacity of liraglutide. Interestingly, these changes occurred independently of glycemic control, which gives hope for improving cardiovascular risk in patients.

A more recent study compared the effects of liraglutide, sitagliptin, and diet on inflammation and endothelial function, among other things. Endothelium was assessed by measuring flow-mediated vasodilation. A measure of inflammation was the concentration of the chemokine monocyte chemoattractant protein-1 (MCP-1) [28], levels of which are increased by oxidative stress or endothelial dysfunction [29,30]. Liraglutide (as well as the other applied treatments) has been shown to have no effect on endothelial dilatation function. In contrast, it has been suggested that improved flow-mediated vasodilation is achieved in patients with significant endothelial dysfunction. Liraglutide, regardless of its effect on body weight, reduced MCP-1, thus proving its anti-inflammatory properties [28].

The studies described above are presented and compared in Table 3 [26,28].

## 5. Microvascular Outcomes of Liraglutide Treatment

Although the incidence of microvascular complications is decreasing [31], they increase the risk of major adverse cardiovascular events (MACE) and multiple organ complications [32]. Studies on the effects of liraglutide on microvascular outcomes include microvascular complications, such as nephropathy [33,34], retinopathy [34], or neuropathy [33], as well as effects on coronary microvascular function [35,36].

The GRADE Study Research Group conducted a comparison of the effects of adding insulin glargine U-100, glimepiride, liraglutide, and sitagliptin to metformin treatment. They focused on cardiovascular and microvascular outcomes. Microvascular complications were defined as diabetic peripheral neuropathy and increased albuminuria or eGFR <60 mL/1.73 m^2^. Liraglutide was not observed to reduce the incidence of microvascular disease more effectively than other hypoglycemic drugs tested. It also did not attenuate declines in renal function; however, a lower risk of moderate albuminuria was observed in the sitagliptin and liraglutide groups [33].

Previous studies indicated that liraglutide, by acting as a nephroprotective agent [37], protects against the development and progression of nephropathy [38]. The LEADER study, conducted by Marso et al. [34], showed that taking liraglutide results in a lower risk of microvascular events when compared to placebo. In this study, microvascular events were defined as the occurrence of nephropathy or retinopathy [34]. This is the effect of a reduction in the incidence and progression of nephropathy, despite an increase in the occurrence of retinopathy, which was found to be statistically insignificant [34,38]. A similar conclusion regarding the effect of liraglutide use on renal outcome is provided by a meta-analysis conducted by Cha et al. [39]. Interestingly, Verma et al. [32], based on an analysis of the LEADER trial, report that the burden of microvascular complications increases the risk of MACE, while the use of liraglutide reduces the risk.

Studies focusing on, among other things, the effect of liraglutide therapy on the occurrence of nephropathy and/or retinopathy and/or neuropathy are presented in Table 4 [33,34].

Interesting studies were also conducted on, among other things, the effect of liraglutide on the microvascular function of the coronary vessels (Table 5.) [35,36]. A study by Suhrs et al. [35] focused on the effect of liraglutide on microvascular dysfunction as it increases CV risk. The measure of microvascular function was coronary flow velocity reserve (CFVR) and the severity of angina symptoms. Unfortunately, despite improvements in body weight, blood pressure, and lipid levels, the treatment administered was not shown to affect coronary microvascular function [35]. It has been suggested that the result of the study was influenced by the fact that the measurement of CFVR took place 1 to 2 weeks after the administration of liraglutide, so the direct effect of the drug on coronary microcirculation was not assessed [35,40]. A study by Faber et al. [36] provides consistent conclusions. With concomitant reductions in body weight, systolic blood pressure, and HbA1c levels, there was no effect of liraglutide treatment on coronary microvascular function, which was assessed using non-invasive Doppler echocardiography under dipyridamole-induced stress. However, the probable reason for the lack of the expected therapeutic effect is the too short duration of the study [36]. Thus, liraglutide therapy did not improve coronary microcirculation in both diabetic and non-diabetic patients; however, further studies are required [35,36]. This is interesting in the context of reports on the effect of weight loss on microcirculatory outcomes [41,42,43]. Coppola et al. [41] described a correlation between weight loss, adiponectin concentrations, and improved CFVR. Similarly, in a study by Nerla et al. [42], one of the effects of bariatric surgery appeared to be an improvement in coronary microcirculation via improved peripheral endothelial function. Moreover, this effect has been shown to be long-lasting [43].

Smits et al. [44] conducted a study in which microvascular perfusion was tested to assess the effect of liraglutide on microvascular outcomes. For this purpose, nail skin capillary videomicroscopy and laser Doppler fluxometry were used. Capillary perfusion is affected by many mediators, such as nitric oxide or insulin. Fifty-five T2DM patients were enrolled, nineteen of whom received 1.8 mg liraglutide per day. Fasting or postprandial measurements were taken to determine microvascular function. The RCT lasted 12 weeks. No improvement in microvascular perfusion was observed. This result is explained, among other factors, by the fact that endothelial dysfunction occurs in patients suffering from T2DM. In addition, treatment with liraglutide had no effect on vasomotion. 

## 6. Cardiovascular Outcomes of Liraglutide Treatment

Patients with T2DM are at particular risk for adverse cardiovascular events. GLP-1 agonists are not only hypoglycemic drugs, but have also been shown to have beneficial effects on reducing cardiovascular risk, the reduction of which is so important in this group of patients [45]. 

Kristensen et al. [45] conducted a meta-analysis evaluating the effects of GLP-1 receptor agonists on cardiovascular outcomes in patients with T2DM. In six of the seven included studies, the rate of MACE, which included cardiovascular death, stroke, and non-fatal MI, was reduced by 12%. In addition, deaths from all causes and hospital admissions for heart failure were reduced [45]. The same problem was addressed by Bethel et al. [46] in their meta-analysis, which focused on four large trials: LEADER, ELIXA, EXSCEL, and SUSTAIN 6; when compared to placebo for three-point MACE, a 10% relative risk reduction was observed in all trials. In addition, there was a 13% reduction in mortality from cardiovascular causes and 12% from any cause. However, no significant difference was seen with placebo for fatal and non-fatal stroke or MI, as well as hospitalizations for heart failure and unstable angina [46]. In a meta-analysis by Zhu et al. [13], an overall ability to reduce adverse cardiovascular events, including death from them, stroke, or MI, as well as atrial fibrillation and heart failure, was noted in the GLP-1 receptor agonist group. Some effects were specific to the drugs in question, such as a reduction in the rate of MI for abiglutide and a reduction in stroke for semaglutide and dulaglutide. It has been reported that the chemical structure of drugs can affect the protective capacity against cardiovascular events, with the highest capacity being characterized by drugs based on human GLP-1, including liraglutide [13]. In turn, Tsapas et al. [15] balanced the benefits and harms of hypoglycemic treatment in patients with T2DM and showed that patients using liraglutide with high cardiovascular risk had a lower risk of cardiovascular mortality, while dulaglutide and subcutaneous semaglutide reduced the risk of stroke. In contrast, there were no significant differences in MI among all groups. In untreated patients and those at low cardiovascular risk, there were no significant differences between drugs in reducing cardiovascular outcomes as there were in patients already treated with metformin; however, in these patients, the addition of liraglutide was most effective in reducing HbA1c levels [15]. The GRADE randomized trial focused on the efficacy of metformin combination therapy with other hypoglycemic drugs; it showed that adding liraglutide or glargine to metformin had a better effect in lowering HbA1c. The liraglutide group had the lowest incidence of hypertension and also the lowest mean systolic blood pressure. Moreover, in this group, a negligible number of patients experienced any cardiovascular incident during the first year of the study, reaching 10% at the end of the study, compared to 14% for other drugs. Mortality from cardiovascular causes or any cause was similar in all groups. Overall, patients in the current study had a lower cardiovascular risk, so the positive results with regard to cardiovascular events may suggest a protective effect of GLP-1 receptor agonists in this group of patients; however, such conclusions should be made with caution and not taken as definitive evidence [33].

In a RCT conducted by Marso et al. [34], patients using liraglutide with high cardiovascular risk and with T2DM were studied; a lower rate of death from cardiovascular causes and death from any cause was noted. At the same time, a reduced rate of stroke and non-fatal MI was noted; however, these results were not statistically significant [34]. In contrast, the RCT conducted by Gilbert et al. [47] focused on older patients with T2DM from the LEADER trial, including patients 75 years of age or older, as well as patients 60–74 years of age with risk factors for CVD, and patients younger than 60 years of age but with CVD. Elderly patients aged 75 years or older have been shown to benefit more from liraglutide treatment in reducing MACE and death from any cause when compared to younger patients; however, a smaller number of patients in the older group were studied and the follow-up time was quite short [47]. The purpose of the randomized trial conducted by Buse et al. [48] was to identify the factors that determined the improvement in cardiovascular risk in patients taking liraglutide in the LEADER trial. It appears that HbA1c may be a factor contributing to the effectiveness of liraglutide therapy in reducing cardiovascular complications. It is also possible that there are other mediators of liraglutide treatment, such as body weight and hypoglycemia, but their role is uncertain and requires further study [48]. 

Patients with T2DM who have already developed complications of atherosclerotic CVD like MI or stroke are at significant risk for further adverse events of this type. The purpose of the RCT conducted by Verma et al. [49] was to perform a post-hoc analysis of the LEADER trial and evaluate the efficacy of liraglutide in patients with T2DM and with or without a history of stroke or MI. It showed that patients with a history of stroke or MI had the highest risk of MACE (18.8%), followed by those without stroke or MI but with established atherosclerotic CVD (11.6%); those with only cardiovascular risk factors had the lowest risk (9.8%). It was observed that the patients using liraglutide benefited on all cardiovascular endpoints when compared to placebo; however, in patients with cardiovascular risk factors only, liraglutide had a neutral effect on outcomes. Studies with larger numbers of subjects and longer durations are needed to assess whether liraglutide definitively has a neutral effect in patients with cardiovascular risk factors only [49].

Because of the beneficial effects of GLP-1 analogs in animals in reducing myocardial necrosis after MI, there has been interest in whether a similar phenomenon might occur in humans. A study by Nauck et al. [50] analyzed and assessed the risk of a composite endpoint, including hospitalization for heart failure or risk of death from cardiovascular causes in post-MI patients from the LEADER trial. Patients after MI were shown to have a seven-fold higher risk of the events in question than patients without a history of MI; however, there was no significant difference in the reduction of the composite endpoint in post-MI patients after liraglutide versus placebo. Moreover, no differences in the cardioprotective effect of liraglutide were also observed between patients without and after MI [50].

Patients with T2DM are at greater risk of developing heart failure, causing left ventricular diastolic dysfunction. Bizino et al. [51] set out to evaluate how liraglutide affects cardiac function among patients with T2DM. In this 26 week study, patients with diabetic cardiomyopathy but no prior CVD were assigned to a group receiving 1.8 mg liraglutide or placebo; the effect of treatment was assessed by cardiac magnetic resonance imaging. A significant improvement in diastolic function parameters was observed in the liraglutide group; however, no significant difference was noted for systolic function parameters, such as volume and ejection fraction. Liraglutide reduced left ventricular filling pressure, which may positively translate into left ventricular function and delay the progression of heart failure. It is noteworthy that liraglutide decreased left ventricular function parameters like ejection fraction; however, they were still within the normal range [51]. A comparison of the cardiovascular outcomes of some of the studies discussed is summarized in Table 6 [34,47,49,50].

## 7. The Use of Liraglutide in the Treatment of Obesity—Results

Obesity is a global problem, affecting people of all ages and from different regions of the world, both developing and developed countries. It is associated with an increased risk of various diseases, including diabetes or hypertension, but also causes a reduced quality of life. Due to its mechanism of action, liraglutide can be an effective adjunctive therapy for obesity [52,53]. 

Zhang et al. [52] decided to study the efficacy and safety of liraglutide in obese people without diabetes. They proved that, when compared to placebo, a dose of 3.0 mg per day resulted in a greater reduction in mean body weight, as well as a higher percentage of 5% weight loss. Liraglutide was shown to be well tolerated by patients, although it was more likely to cause nausea when compared to placebo [52]. The SCALER study also examined the effect of liraglutide at a dose of 3.0 mg for this reason, but in patients with T2DM treated with insulin and hypoglycemic drugs. After 56 weeks of the study, mean body weight in the liraglutide group was reduced by 5.8% when compared to placebo (1.5%). A similar relationship was seen in the percentage of patients with weight loss of >5%, where it was recorded at 51.8% in the liraglutide group [54]. Similarly, Konwar et al. [53] focused on the efficacy and safety of liraglutide 3.0 mg among obese patients both with and without T2DM. At 52 weeks, there was a significant reduction in mean body weight, mean body mass index (BMI), waist circumference, and the percentage of patients with a 5% reduction in body weight when compared to placebo. The liraglutide group had a higher rate of adverse events; however, serious adverse events were similar in both groups, so liraglutide appears to have a favorable safety profile [53]. Kelly et al. [55] examined the effect of liraglutide therapy in adolescents with obesity. After 56 weeks, they observed that liraglutide combined with diet showed a better effect in reducing the standard deviation in BMI, as well as in the percentage of patients with BMI reductions >5% and >10%. The liraglutide group had a higher incidence of gastrointestinal adverse events, so this therapy may not be appropriate for all patients [55]. Zhou et al. [56] focused on another aspect and set out to determine the factors most influencing weight reduction in obese or overweight patients with T2DM among the Chinese population using liraglutide. Interestingly, rather than baseline BMI and waist circumference values, it was the severity of side effects during liraglutide therapy proved to be the greatest predictor of BMI and waist circumference reduction. Of the side effects, nausea and anorexia were the most common, while bloating, diarrhea, and vomiting were observed less frequently. It was shown that in patients with good tolerance, side effects resolved after about 3.1 weeks, and the weight reduction effects persisted after they stopped [56]. Table 7 summarizes the effect of liraglutide on weight reduction from the papers described above [52,53,54,55,56]. 

## 8. Conclusions

T2DM is a common disease and the number of new cases is constantly increasing. This metabolic disorder is based on impaired B-cell secretion and tissue resistance to insulin. Despite numerous anti-diabetic drugs, maintaining target blood glucose levels and protecting against adverse side effects, including hypoglycaemic episodes, can be very difficult. Liraglutide, which is a GLP-1 receptor agonist, seems to be a promising drug that both regulates the level of glycemia and affects cardiovascular risk; it may reduce the number of deaths and, what is more, it causes weight loss with additional benefits. The current study assessed its effectiveness and safety in the treatment of T2DM, providing evidence that it is a well-tolerated drug that does not cause many side effects, and is, at the same time, effective. 

Liraglutide alleviates hyperglycemia-induced endothelial dysfunction. It demonstrated its oxidative stress-reducing properties by reducing ROS, lipid hydroperoxides, and heme oxygenase-1, while preventing apoptosis by an increase in the concentration of anti-apoptotic protein Bcl-2 and a decrease in the level of pro-apoptotic protein Bax. A measure of the effect on inflammation was a decrease in the concentration of MCP-1. Liraglutide also restored GLP-1R/PKA and GTPCH1/eNOS pathway signaling, and through the PI3K/Akt/survivin pathway. appeared to restore calcium homeostasis. In conclusion, liraglutide shows antioxidant and anti-inflammatory properties in a mechanism independent of glycemic control or weight loss.

Liraglutide has not been observed to affect the incidence of microvascular complications more effectively than glargine, glimepiride, or sitagliptin. However, treatment with liraglutide has been proven to reduce the occurrence and progression of nephropathy. In both diabetic and non-diabetic patients, liraglutide did not improve coronary microvascular function; however, the study authors indicate that further studies are needed.

Liraglutide appears to be an effective predictor of cardiovascular events by reducing mortality from cardiovascular causes or any cause, as well as preventing the occurrence of non-fatal strokes and myocardial infarctions. Interestingly, it appears that higher-risk patients may benefit most from liraglutide treatment.

GLP-1 receptor agonists are safe drugs whose benefit-to-risk ratio means that these drugs should be widely used in patients with T2DM to reduce cardiovascular events. However, it is worth noting that hypoglycaemic therapy should be selected individually for a given patient, assessing the risk profile and benefits of therapy.

This paper was intended to familiarize the reader with the mechanism of action of liraglutide, its beneficial effects on the microvascular and cardiovascular systems, and its effectiveness in reducing body weight in patients with type 2 diabetes. 

## Figures and Tables

**Table 1:** Classification of GLP-1 receptor analogues [8,9].

Long-Acting	Short-Acting
Liraglutide	Exenatide (administered twice a day)
Exenatide (administered once a week)	Lixisenatide
Dulaglutide	
Semaglutide	

**Table 2:** Comparison of efficacy and safety of GLP-1 receptor agonists from the described studies [7,17,18,19].

**Authors**	Htike et al. [7]	Taheri et al. [17]	Seino et al. [18]	Wang et al. [19]
**Year**	2017	2019	2022	2021
**All patients**	14,464	1433	466	1557
**Study design**	Systematic review	Systematic review and metaanalysis	RCT	Meta-analysis
**Patient characteristics**	Obese or overweight patients with metabolic syndrome aged 35 and over	Patients with T2DM	Patients with T2DM	Patients with T2DM and CAD
**Duration**	40 weeks	26–52 weeks	26 weeks	8–26 weeks
**Safety and efficacy in lowering blood glucose levels**	GLP-1R analogues are effective drugs in glycemic control. A higher incidence of gastrointestinal adverse events has been demonstrated	Liraglutide and Dulaglutide effectively reduced HbA1c levels, with Dulaglutide showing greater efficacy in this regard. Dulaglutide was more likely to cause gastrointestinal adverse effects	Increasing the dose of liraglutide from 0.6 mg to 1.8 mg once a day was more effective in lowering HbA1c levels. Both doses were well tolerated by patients.	The inclusion of liraglutide in therapy significantly improved indicators of glycemic control including lowered HbA1c, FBG. It probably did not increase the risk of side effects.

RCT, randomized controlled trial; T2DM, type 2 diabetes mellitus; CAD, coronary artery disease; GLP-1R, glucagon-like peptide-1 receptor; HbA1c, glycated hemoglobin; FBG, fasting blood glucose.

**Table 3:** Comparison of studies on the effect of liraglutide treatment on oxidative stress, inflammation, and/or endothelial dysfunction [26,28].

**Authors**	Rizzo et al. [26]	Mashayekhi et al. [28]
**Year**	2015	2023
**All patients**	20	88
**Study design**	clinical trial	RCT
**Patient characteristics**	T2DM	Obesity prediabetes
**Aim of the study**	evaluation of the effects of liraglutide on oxidative stress, HO-1, and ghrelin levels	evaluation of the effects of GLP-1R agonists on vascular endothelial function, fibrinolysis, and inflammation
**Duration**	2 months	14 weeks
**Treatment with liraglutide**	initial dose 0.6 mg per day, after two weeks 1.2 mg per day	initial dose 0.6 mg per day, after one week 1.2 mg per day, after two weeks 1.8 mg per day
**Effect of liraglutide on oxidative stress/inflammation/endothelial dysfunction**	decrease in serum levels of lipid hydroperoxides and HO-1	reduction in chemokine MCP-1 levels

HO-1, heme oxygenase-1; MCP-1, chemokine monocyte chemoattractant protein-1.

**Table 4:** Comparison of studies on the effect of liraglutide treatment on microvascular complications [33,34].

**Study**	GRADE	LEADER
**Authors**	GRADE Study Research Group [33]	Marso et al. [34]
**Year**	2022	2016
**All patients**	5047	9340
**Study design**	RCT	RCT
**Patient characteristics**	T2DM (diagnosed in the past 10 years and treated with ≥500 mg of metformin daily HbA1c 6.8–8.5%	T2DM high CV risk
**Aim of the study**	Comparison of glucose-lowering drugs in relation to microvascular and CVD outcomes.	To investigate the CV effect of liraglutide therapy.
**Duration**	5.0 years	3.8 years
**Treatment with liraglutide**	1.8 mg per day	1.8 mg per day
**Microvascular outcome**	no significant differences in the incidence of microvascular complications between the glargine, glimepiride, liraglutide, and sitagliptin groups	lower risk of microvascular events

CVD, cardiovascular disease; CV, cardiovascular.

**Table 5:** Comparison of studies on the effect of liraglutide treatment on coronary microvascular function [35,36].

**Authors**	Suhrs et al. [35]	Faber et al. [36]
**Year**	2019	2015
**All patients**	29	20
**Study design**	RCT	RCT
**Patient characteristics**	woman non-diabetic BMI > 25 CMD (CFVR ≤ 2.5) angina symptoms no obstructive CAD	T2DM no history of CAD
**Aim of the study**	Verifying whether liraglutide ameliorates CMD and symptoms through weight loss.	To examine the short-term effects of GLP-1 therapy on coronary microcirculation.
**Duration**	control period: 5 weeks intervention period: 12 weeks	treatment: 10 weeks wash-out: 2 weeks
**Treatment with liraglutide**	3 mg per day	initial dose 0.6 mg per day, after two weeks 1.2 mg per day
**Microvascular outcome**	the lack of improvement in coronary microvascular function	no significant effect on microvascular function

BMI, body mass index; CMD, coronary microvascular dysfunction; CFVR, coronary flow velocity reserve.

**Table 6:** Comparison of studies on the effect of liraglutide treatment on cardiovascular outcomes [34,47,49,50].

**Authors**	Marso et al. [34]	Gilbert et al. [47]	Verma et al. [49]	Nauck et al. [50]
**Year**	2016	2019	2018	2018
**All patients**	9340	9340	9340	9340
**Study design**	RCT	RCT	RCT	RCT
**Patient characteristics**	Patients with T2DM and high CV risk	Patients aged 75 years or older, patients aged 60–74 years with risk factors for CVD, and patients younger than 60 years but with CVD	Patients with or without T2DM and MI/stroke	Patients with T2DM and high CV risk
**Duration**	3.8 years	3.8 years	3.8 years	3.8 years
**Cardiovascular outcome**	There was a lower rate of death from CV causes and death from any cause among individuals using liraglutide.	Elderly patients aged 75 years or older benefited more from liraglutide treatment in reducing MACE and death from any cause compared to younger patients	Patients on liraglutide benefited on all CV endpoints compared to placebo, but in patients with CV risk factors only, liraglutide had a neutral effect on outcomes.	Patients with a history of MI had a 7 times greater risk of hospitalization for heart failure or risk of death from CV causes than patients without a history of MI.

MI, myocardial infarction; MACE, major adverse cardiovascular events.

**Table 7:** Comparison of studies on the effect of liraglutide treatment on weight reduction [52,53,54,55,56].

**Authors**	Zhang et al. [52]	The SCALE study [53]	Konwar et al. [54]	Kelly et al. [55]	Zhou et al. [56]
**Year**	2019	2020	2022	2020	2022
**All patients**	4754	396	6867	251	90
**Study design**	Meta-analysis	RCT	Systematic review and meta-analysis	RCT	RCT
**Patient characteristics**	Obese patients without T2DM	Overweight or obese patients with T2DM using basal insulin	Overweight or obese patients with or without diabetes	Adolescents with obesity	Overweight or obese patients with T2DM
**Aim of the study**	Safety and efficacy of liraglutide in obese patients without diabetes mellitus	Evaluation of the efficacy and safety of incorporating liraglutide into insulin therapy in obese or overweight patients with T2DM	Evaluation of the efficacy and safety of liraglutide in obese or overweight patients with or without diabetes	Safety and efficacy of liraglutide in obese adolescents	Identification of factors having the greatest impact on weight loss in patients using liraglutide
**Duration**	14–55 weeks	52 weeks	12–56 weeks	56 weeks	52 weeks
**Treatment with liraglutide**	3.0 mg once a day	3.0 mg once a day	3.0 mg once a day	3.0 mg once a day	1.2 mg or 1.8 mg once a day
**Impact on body weight**	Weight reduction	Weight reduction	Weight reduction	Weight reduction	Weight reduction

## Data Availability

The data used in this article are sourced from materials mentioned in the References section.
